# Vehicle Detection Algorithms for Autonomous Driving: A Review

**DOI:** 10.3390/s24103088

**Published:** 2024-05-13

**Authors:** Liang Liang, Haihua Ma, Le Zhao, Xiaopeng Xie, Chengxin Hua, Miao Zhang, Yonghui Zhang

**Affiliations:** 1 College of Information Science and Engineering, Henan University of Technology, Zhengzhou 450001, China; liangliang@stu.haut.edu.cn (L.L.); lezhao@haut.edu.cn (L.Z.); xiaopengxie@stu.haut.edu.cn (X.X.);; 2 Key Laboratory of Grain Information Processing and Control of Ministry of Education, Henan University of Technology, Zhengzhou 450001, China

**Keywords:** autonomous driving, vehicle detection, sensors, sensor fusion, deep learning

## Abstract

Autonomous driving, as a pivotal technology in modern transportation, is progressively transforming the modalities of human mobility. In this domain, vehicle detection is a significant research direction that involves the intersection of multiple disciplines, including sensor technology and computer vision. In recent years, many excellent vehicle detection methods have been reported, but few studies have focused on summarizing and analyzing these algorithms. This work provides a comprehensive review of existing vehicle detection algorithms and discusses their practical applications in the field of autonomous driving. First, we provide a brief description of the tasks, evaluation metrics, and datasets for vehicle detection. Second, more than 200 classical and latest vehicle detection algorithms are summarized in detail, including those based on machine vision, LiDAR, millimeter-wave radar, and sensor fusion. Finally, this article discusses the strengths and limitations of different algorithms and sensors, and proposes future trends.

## 1. Introduction

The advancement in technology is gradually permeating into the lives of people, with autonomous driving being at the forefront. In particular, autonomous vehicles (AVs) can eliminate 94% of road accidents caused by human error and distracted driving [1]. Against this backdrop, automated driving systems (ADSs) have emerged with the promise of preventing accidents, reducing emissions, transporting the mobility-impaired, and taking the stress out of driving [2]. Autonomous vehicles can be classified into six levels based on the degree of human intervention and attention required, denoted as L0 to L5, each signifying different degrees of autonomy [3]. Currently, most IVs can only achieve partial autonomous driving functions, such as lane-keeping, intelligent speed limitation, adaptive cruise control, etc. The realization of fully automated driving still has a long way to go.

Autonomous driving systems typically consist of three components: environmental perception, behavioral decision making, and motion planning and control [4]. Environmental perception serves as the prerequisite and foundation of autonomous driving [5]. In particular, robust and reliable vehicle detection has been a topic of great interest [6]. Vehicle detection, the ability of a vehicle to perceive its surrounding vehicles in real-world driving scenarios, holds critical importance across various domains, including intelligent transportation, military defense, security surveillance, and autonomous driving [7]. According to traffic accident statistics, the main threat to drivers often comes from other vehicles [8]. Therefore, the efficient sensing and accurate recognition of the surrounding environment are paramount for ensuring the safety of self-driving vehicles. In order for autonomous vehicles to function effectively, they must be aware of other vehicles in a timely manner, allowing them to formulate safe and reliable plans [9]. Given the potential for closing speeds between vehicles, this necessitates the ability to accurately detect vehicles.

Moreover, the performance of vehicle detection directly influences the quality of decision making and control in autonomous vehicles. Detecting vehicles using different sensors is a significant challenge due to the various characteristics of vehicles, such as size, occlusion, orientation, and shadows [10]. Additionally, the time-sensitive nature of vehicle detection, requiring faster processing than other applications, further complicates the task [11]. Therefore, precise vehicle detection is crucial for the automation and intelligence of vehicles. With the rapid development of deep learning, sensor technologies, and the Internet of Things (IoT), more and more new methods and technologies have emerged and are gradually being applied in the field of vehicle detection [12,13]. This paper focuses on sensors and summarizes more than 200 classical and latest vehicle detection algorithms in recent years. This paper also analyzes the tasks, evaluation metrics, and existing public datasets for vehicle detection and presents the future trends of vehicle detection.

The rest of this article is organized as follows: Section 2 describes the tasks, evaluation metrics, and existing public datasets for vehicle detection. Then, Section 3 introduces vision-based vehicle detection algorithms, focusing on the application of deep learning methods. Next, vehicle detection methods based on radar and LiDAR are delineated in Section 4. Section 5 provides an integrated analysis of Section 3 and Section 4, encompassing the implementation of various sensor fusion techniques. Section 6 discusses the different sensors and algorithms for vehicle detection, and offers future trends. Finally, Section 7 is the conclusion.

## 2. Preliminaries for Vehicle Detection

Intelligent vehicles provide drivers with information regarding safety, assistance, and comfort. In environmental perception, demands arise in complex road scenarios to detect and assess various targets in real time and evaluate the effectiveness of detection indicators. This section mainly describes the detection tasks and metrics for intelligent vehicles and introduces some public datasets for vehicle detection.

### 2.1. Tasks

Vehicle detection is of crucial importance in the environment perception framework of intelligent vehicle systems. It facilitates positioning and classifying diverse entities, including pedestrians, non-motorized vehicles, traffic signage, and lane demarcations within road environments. Vehicle detection is divided into 2D object detection and 3D object detection, and both of them are widely applied in vehicle detection tasks. Two-dimensional object detection serves as a fundamental technique in the realm of vehicle detection. It entails utilizing a 2D bounding box within the visual field of intelligent vehicles to select detected objects, and then the selected objects are classified and positioned. The 3D object detection system displays the specific position of the vehicles in the camera coordinate system. This process requires 3D bounding boxes to select the detected objects, followed by their classification and localization [14].

### 2.2. Evaluation Metrics

Metrics such as Intersection over Union (IoU), precision (P), recall (R), F1-score, Average Precision (AP), and mean Average Precision (mAP) are commonly utilized to evaluate detection accuracy of algorithms. IoU quantifies the degree of overlap between predicted bounding boxes and ground truth bounding boxes. Precision indicates the proportion of predicted positive samples that are true positives, whereas recall signifies the proportion of actual positive samples that are correctly identified. F1-score offers a combined measure of precision and recall. These metrics are computed using specific formulas:

(1) $$IoU=\frac{p_{b}\cap g_{b}}{p_{b}\cup g_{b}}$$

(2) $$P=\frac{TP}{TP+FP}$$

(3) $$R=\frac{TP}{TP+FN}$$

(4) $$F1=\frac{2\times P\times R}{P+R}$$

where $p_{b}$ and $g_{b}$ represent predicted boxed and ground truth boxes, respectively; TP denotes the count of positive cases accurately identified as true samples; FP indicates the tally of negative cases erroneously identified as true samples; and FN represents the number of positive cases mistakenly classified as false samples. Additionally, AP measures the precision performance for an individual class, while mAP offers the mean of AP values across all classes. These metrics are formulated using the following equations:

(5) $$AP=\int_{0}^{1}P(R)dR$$

(6) $$mAP=\frac{1}{n}\sum_{i=1}^{n}P(i)\Delta R(i)$$

where n is the number of categories in detection targets.

In the realm of assessing detection speed, parameters, FLOPs (Floating Point Operations), and FPS (Frames Per Second) are essential metrics. Parameters denote the total count of trainable parameters involved in the model training, often used to gauge the size of the model. FLOPs quantify the model’s complexity. The lower value of FLOPs indicates reduced computational load for model inference. FPS assesses the detection speed by indicating the number of frames processed per second.

### 2.3. Datasets

Datasets are indispensable for training models in each task of autonomous driving. High-quality annotated data, such as images, videos, and sensor data from various scenarios, are the basis for training autonomous driving systems and machine learning models, and are manually labeled to indicate information about correct behavior, object detection, and environment perception. In order to propel and invigorate the field of autonomous driving, industry organizations and researchers have produced many high-quality datasets. Table 1 summarizes some essential information from these datasets. We list a number of items, including year, location, scene, category, annotation, 3D boxes, and application scenarios.

## 3. Vehicle Detection Algorithms Based on Machine Vision

Machine vision systems are considered a promising research field with broad applications in various detection scenarios. Machine-vision-based sensors are the earliest and most widely used sensors for vehicle detection [37]. These types of sensors are typically referred to as passive sensors since they solely capture images of the objects without the need for specialized illumination projection devices. Vision-based sensors typically have access to a rich set of perception information from the traffic environment, such as textures, colors, lane markings, obstacle identifications, and semantics. In the past few decades, the rapid development of computer information and sensor technology has led to the widespread adoption of sensor combinations based on multiple visual modalities. For example, companies like Tesla [38] and Mobileye [39] have embraced pure vision solutions for intelligent vehicle environment perception. According to the different principles of existing algorithms, vehicle detection techniques based on machine vision can be categorized into three components: traditional-based, machine learning-based, and deep learning-based techniques [4].

### 3.1. Traditional-Based Methods for Vehicle Detection

Inherent appearance features of vehicles can be exploited in traditional-based vehicle detection methods that typically include two main steps: hypothesis generation (HG) and hypothesis verification (HV). In the HG stage, the system extracts a region of interest (ROI) based on the appearance features of the detected vehicle. And then in the HV stage, the system confirms whether the ROI contains vehicle targets. In other words, HG is the backbone of the process, while HV is the further verification and validation of the generated hypotheses, both of which are necessary. Depending on the traffic scenarios, the appearance features of vehicles can generally be categorized into the following six common types.

- Color: Due to the continuity and concentration of the color distribution of the vehicles in the image, the vehicles can be separated from the image background by applying different color channels and setting appropriate segmentation thresholds [40,41]. However, techniques based on color features are susceptible to variations in illumination and specular reflections [42].
- Symmetry: Most cars have symmetrical rear ends. By leveraging this feature, we can search for regions with high symmetry on ROI in the image to obtain vehicle information, resulting in the identification of vehicle objects and non-vehicle objects. Moreover, symmetry can not only help to optimize the bounding boxes of vehicles, but also be employed to confirm if the ROI includes targets for vehicles in the HV stages [43]. However, the computation of symmetry increases the overhead of time and reduces detection efficiency.
- Edges: Vehicle features such as silhouettes, bumpers, rear windows, and license plates exhibit strong linear textures in both vertical and horizontal directions [44]. Extracting these typical edge features from the image allows for a further determination of the car’s bounding box [45,46]. However, the edge lines may tend to overlap with some texture lines of the image background, which may lead to the appearance of false positives in particular scenes.
- Texture: Typically, road textures exhibit a relatively uniform distribution, whereas textures on car surfaces tend to be less uniform due to the presence of highly varied regions. We can indirectly perform vehicle detection by distinguishing the difference between these two conditions [47]. However, relying on feature textures to detect vehicles may result in low detection accuracy.
- Shadows: In bright daylight, the vehicles traveling on the road cast stable shadows underneath. The shadowed region clearly exhibits a lower gray value compared to the remaining road areas. Utilizing segmentation thresholds enables the extraction of the underlying shadow as the ROI for vehicles during the HG stage [48,49]. However, the application scenarios of this approach are relatively limited.
- Taillights: Vehicle detection at night is often achieved through the use of taillight features due to the noticeable color (usually red). It is easy to extract information from it through image processing techniques [50,51]. However, this method is only effective for detecting vehicles at night.

Traditional vehicle detection methods have the advantages of a low cost, a fast detection speed, and simple working principles. However, these methods are based on prior knowledge and hence are mainly susceptible to interference from other objects. An effective approach is to use the fusion of multiple features for detection [52]. In addition, the extracted ROI can be used as basic feature information and then modeled using machine learning or deep learning methods [48,53].

### 3.2. Machine Learning-Based Methods for Vehicle Detection

With the rapid development of computer technologies, machine learning (ML) has become a popular issue in the realm of vehicle detection. ML, an essential branch within the fields of artificial intelligence (AI) and computer science, is dedicated to using data and algorithms to emulate how humans learn. An ML model transforms and encodes vehicle images using manually designed features and applies a particular mapping method to convert high-dimensional image space data to low-dimensional image space data. The model is then trained continuously to receive a final model for vehicle detection. Typically, vehicle detection using machine learning models can be divided into two key steps: first, the input image is processed to obtain the ROI; second, the extracted image features are fed into a classifier for training and optimization.

#### 3.2.1. Feature Extraction

Ease to extract and identify, while preserving stable vehicle characteristics when the vehicle attitude and type change, is a necessary quality of an effective feature extraction technique. The histogram of oriented gradients (HOG) is one of the popular methods for feature extraction in the field of object detection. It initially achieved significant success in pedestrian detection [54], and has since expanded to other application domains, such as vehicle detection and face recognition. Many researchers have improved upon the HOG algorithm, such as two HOG vectors [55], the pyramid of HOG [56], and symmetry HOG [57]. The deformable part model (DPM) employs the improved HOG descriptor and adopts a multi-component strategy [58]. The Haar-like vector is also a fundamental descriptor used for face detection and was later extended to vehicle detection [59]. Some other feature extraction methods are also frequently used for vehicle detection, such as a local binary pattern (LBP) [60], Gabor filters [61], and sped-up robust features (SURFs) [62]. Moreover, some studies have shown that the fusion of multiple feature descriptors may result in a richer representation of vehicles [63,64].

#### 3.2.2. Classifier

An ML classifier can distinguish vehicle and non-vehicle targets based on the local features collected from the image. In general, a classifier needs to be trained on well-labeled datasets first, with boundaries drawn between positive samples and negative samples. AdaBoost, K-nearest neighbor (KNN), Naive Bayes (NB), Support Vector Machine (SVM), and Decision Tree (DT) are the more commonly used classifiers for vehicle detection. The selection of a classifier requires the consideration of both its generalization ability and fitting accuracy. The generalization ability determines how well the model adapts to new data, while the fitting accuracy measures whether the classifier has a sufficient fit on the training data to identify patterns and associated information accurately. Ensemble learning is a classic machine learning method, combining predictions from multiple base classifiers to enhance overall predictive performance [65,66]. Different studies on feature engineering and classifiers for vehicle detection are shown in Table 2. Vehicle detection methods based on machine learning typically require scanning the entire image to extract features. However, this process increases computational costs and time consumption. Ref. [67] notes that more than half of the image area contains no vehicle information. The use of traditional-based feature extraction combined with a classifier has proved to be an effective approach to address this difficulty. For instance, the ROI for the vehicle was extracted from the image by utilizing the shadow. Then, Haar-like features and the AdaBoost classifier were employed to detect the vehicles from the ROI [55].

### 3.3. Deep Learning-Based Methods for Vehicle Detection

**Figure 1:**
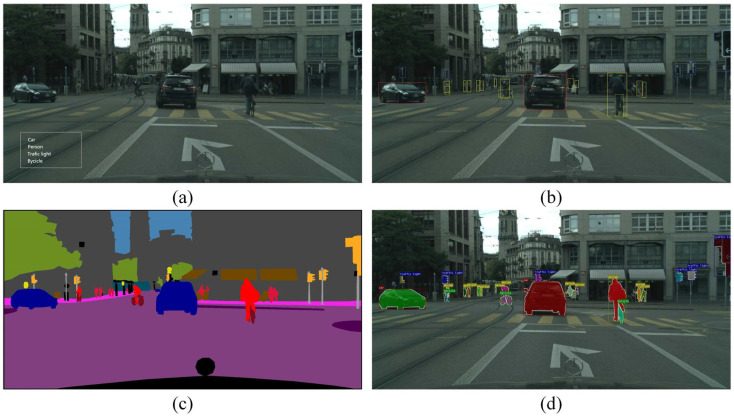
Relationship between different vehicle detection algorithms: (**a**) object classification, (**b**) object detection, (**c**) semantic segmentation, (**d**) instance segmentation [16].

ML-based methods typically rely on manually designed feature extractors and classifiers, which, to some extent, limit the representational capacity of the models. With the rise in deep learning, especially the introduction of convolutional neural networks (CNNs), great progress has been achieved in object detection [73]. Object detection is a pivotal subtask in the field of computer vision, often closely associated with object classification, semantic segmentation, and instance segmentation. Object classification refers to recognizing the different object classes present in an image, while target detection further determines the relative positions of the objects on this basis and locates them by means of bounding boxes. Semantic segmentation is a technique that assigns each pixel to a semantic category label. Instance segmentation, on the other hand, is an extension of semantic segmentation with the goal of distinguishing between different object instances. Figure 1 illustrates the comparison of them. Vehicle detection frameworks under deep learning techniques can be divided into two types: object-detection-based models and segmentation-based models.

#### 3.3.1. Object-Detection-Based Methods

**Figure 2:**
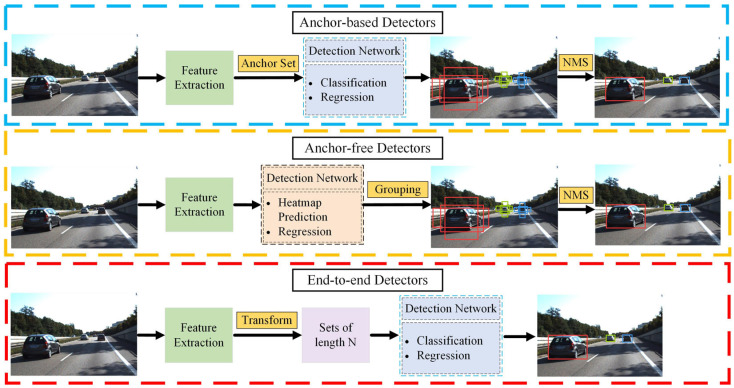
Vehicle detection methods in different detectors [15].

Object detection holds significant potential across diverse applications such as image recognition and video surveillance. In general, object-detection-based models are classified as anchor-based detectors, anchor-free detectors, and end-to-end detectors, as illustrated in Figure 2.

(1) Anchor-Based Detectors

In anchor-based models, predefined bounding boxes are used to detect target objects. Depending on whether region proposals are utilized, anchor-based detectors fall into two types: two-stage and one-stage.

Two-stage: In vehicle detection, vehicle region proposals are first generated followed by classifying and regressing vehicle targets of interest from region proposals. R-CNN series [73,74,75,76], SPP-Net [77], R-FCN [78], FPN [79], and Cascade R-CNN [80] are examples of typical two-stage detectors. Faster R-CNN [73] consists of a separate region proposal network and R-CNN [74] to detect objects, considerably lowering the running time consumed by the detection network. Two-stage methods refine anchors multiple times, resulting in more accurate results compared to one-stage methods.

One-stage: The method predicts the center and bounding boxes of vehicles by placing anchors on the feature map. Typical representatives of one-stage detectors include SSD [81], M2Det [82], RetinaNet [83], and part of the YOLO series [84,85,86,87,88]. YOLOv1 [89] is the pioneering work of the YOLO family of algorithms. As an anchor-free model, it laid the foundation for subsequent YOLO algorithms. From YOLOv2 to YOLOv5, all versions use the anchor-based approach and continue introducing new techniques through each iteration, all of which have improved detection performance. YOLOv4 extensively tests and applies some commonly used tricks in deep learning algorithms to achieve the optimal balance between detection speed and accuracy. YOLOv5 continues the style of the YOLO series of algorithms, and has a strong advantage in the deployment of mobile devices. The innovative YOLOv7 introduces efficient layer aggregation networks (ELANs) as a backbone, and re-parameterized convolutions are employed to accelerate the inference speed. Although one-stage algorithms exhibit lower detection accuracy compared to two-stage algorithms, they hold an advantage in terms of detection speed.

(2) Anchor-Free Detectors

The anchor-free model predicts the center point or keypoints of an object directly and clusters them into a single entity to obtain bounding boxes. The keypoint-based approach involves detecting critical features of the target or the interrelations among these features to determine the target’s position and shape. These critical features may include the corners and center of the object. Some models such as CornerNet [90], RepPoints [91], CenterNet [92], ExtremeNet [93], and Grid R-CNN [94] are keypoint-based. CornerNet identifies an object’s bounding box by detecting a pair of keypoints. CenterNet advances this approach by utilizing a triplet of keypoints instead of a pair. This modification aims to enhance both precision and recall in object detection tasks. The center-based approach determines the target’s bounding box by predicting its center point and positional offset with respect to the center point. Some classical center-based models include YOLOv1 [89], FSAF [95], FCOS [96], GA-RPN [97], FoveaBox [98], YOLOX [99], YOLOv8 [100], and YOLOv9 [101]. FCOS considers all locations within the object bounding box as positives, utilizing four distances and a novel center score for object detection. GA-RPN defines the pixels within the central region of the object as positives, predicting object proposal locations, widths, and heights for Faster R-CNN. Anchor-free detectors are usually more computationally efficient compared to anchor-based detectors. YOLOv8 adopts a novel C2F module that enriches the gradient flow and employs a decoupled head for regression. YOLOv9 introduces generalized ELAN based on YOLOv7 and proposes programmable gradient information to accommodate customized network structures. It is expected for YOLOv9 to become the industry standard for anchor-free detectors in the near future.

(3) End-To-End Detectors

Anchor-based methods rely on proposals or anchors, whereas anchor-free methods utilize center points or keypoints. They indirectly predict a set of bounding boxes by regression and classification tasks. The efficacy of their performances is notably shaped by non-maximum suppression procedures aimed at consolidating near-identical forecasts, by the formulation of anchor sets, and by the heuristics governing the allocation of target boxes to anchors. End-to-end detectors analyze an input image to directly determine the location and category of a target without the need for complicated pre-processing or post-processing procedures. Some models such as DeFCN [102], Sparse R-CNN [103], and DETR [104] are end-to-end-based. DeFCN is based on FOCS [96] and introduces a Prediction-aware One-To-One (POTO) label assignment for classification. Sparse R-CNN re-evaluates the design process of RPN and provides a fixed sparse set of learned object proposals (total length of N) to the object recognition head to perform classification including location. DERT is a new style of neural network based on Transformer [105] for end-to-end detection. Unlike traditional convolutional networks, Transformer-based networks use self-attention mechanisms for encoding and decoding, and can model global feature information. The encoder–decoder architecture was initially proposed for machine translation tasks and has since been widely used in various deep learning models [106]. In vehicle detection, the role of the encoder is to encode the features of input images and map them to high-dimensional vector representations. The decoder is responsible for mapping the encoded features to the output space, which includes categories and positions of the vehicles. DETR transforms the target detection task into an unordered ensemble prediction challenge. It feeds the extracted feature sequences into both the encoder and decoder of the Transformer, yielding an unordered set of length N as output. Each element within the set comprises the object’s category and coordinates. Deformable DETR [107], Anchor-DETR [108], and RT-DETR [109] are also some excellent algorithms based on improved DETR. End-to-end detectors can simplify the vehicle detection process and have an auspicious future.

The Microsoft Common Objects in Context (MS COCO) dataset is widely recognized as one of the most authoritative datasets in the field of object detection. It encompasses 80 object categories, with a total of 2.5 million labeled instances across 328 k images. As a benchmark, we have conducted the performance comparison of various deep models on the MS COCO dataset, as illustrated in Table 3.

#### 3.3.2. Segmentation-Based Methods

Semantic segmentation is generally considered to be more precise and accurate than target-level vehicle detection methods. It attempts to assign a label or category to each pixel in an image and has a greater ability to identify a collection of pixels from different categories and show the position and contour information of vehicles [16], making it important for autonomous vehicles’ environmental perception. Semantic segmentation can be categorized into fully supervised algorithms and weakly supervised algorithms. Weakly supervised learning is a method that utilizes partial, inaccurate, or noisily labeled data for model training [119]. Although this method requires less annotated data and has relatively lower costs, the presence of noise or even mislabeling can impact the accuracy of detection. In autonomous driving, such inaccurate detection significantly affects its performance and safety in real-world scenarios. The fully supervised algorithms are almost always used in most scenarios due to the low security of weakly supervised algorithms.

Traditional vehicle semantic segmentation methods rely on region classification. The principle of these methods is similar to the two-stage detectors, in which vehicle candidate regions are first extracted by a region proposal network, and then a trained classifier assigns labels to each pixel within the candidate regions. DeepMask [120] is a CNN-based model that outputs a class-agnostic segmentation mask, followed by the likelihood score that the patch lies in the center of the vehicles. SharpMask [121] employs a top–down refinement method to generate high-fidelity masks to augment the feed-forward network. MultipathNet [122] makes three improvements on the Fast R-CNN [75] and incorporates DeepMask proposals for detection. Mask R-CNN [76] extends Faster R-CNN [73] to detect different scales and overlapping vehicles in an image with anchor boxes. However, these methods rely on generating candidate regions, which limits the ability to deploy vehicle detection in real time.

The use of pixel-level classification methods helps to improve the issue. A full convolution network (FCN) is a classical algorithm that was first proposed in 2015 [123]. The model replaces the fully connected layers with convolutional layers and uses skip architecture to fuse feature information. SegNet [124] builds on this with an encoder–decoder network. The decoder network maps the low-resolution representation of the encoder to full input resolution feature maps and performs non-linear upsampling in the max-pooling step of the corresponding encoder. Google Labs improved FCN with four separate proposed algorithms. DeepLabv1 [125] introduces the CRF model and atrous convolution to extract image information. DeepLabv2 [126] is built on DeepLabv1 with the backbone of Resnet [127] and an atrous spatial pyramid pooling (ASPP) module. DeepLabv3 [128] combines ideas from DeepLabv1 and DeepLabv2 to segment objects at multiple scales. DeepLab3+ [129] adopts Xception [130] as the backbone and introduces depthwise separable convolution to replace some of the convolutional and pooling layers. DeepLab series can effectively increase the filter’s receptive field. Nevertheless, the Deeplab series requires a high computational cost to deploy in real scenarios.

To further improve the speed and accuracy of vehicle semantic segmentation, some researchers have proposed feature fusion models. These models use multiscale convolution to better access the deep contextual information of an image through a cross-layer structure and reduce computational consumption to some extent. RefineNet [131] efficiently fuses high-level features with finer-grained low-level features to prevent image resolution degradation. PSPNet [132] proposes a pyramid pooling module that exploits global context information by the context aggregation of different regions. ICNet [133] incorporates multi-resolution branches by proper label guidance and introduces the cascade feature fusion unit for fast and high-quality segmentation. In addition, some scholars have attempted to use a generative adversarial network (GAN) for vehicle semantic segmentation [134,135]. However, these methods are unstable during training and fine-tuning, and they are prone to cause the model to collapse and fall into a local optimum.

The Transformer-based architecture is also being applied to semantic vehicle detection as a powerful feature extractor. SERT [136] utilizes ViT [137] as its backbone while integrating multiple CNN decoders to enlarge feature resolution. SegFormer [138] designs a novel hierarchical structured Transformer block to acquire multiscale features and uses MLPs to simply aggregate the features from different layers for decoding. SeaFormer [139] employs a squeeze axial and detail-enhanced attention module to achieve the optimal trade-off between segmentation accuracy and latency on ARM-based mobile devices.

Generally, semantic segmentation-based vehicle detection methods require high computational complexity, which can lead to slower inference speed than that of other vehicle detection algorithms. Therefore, the design and deployment of lightweight models is where the need for the future lies, which requires both speed and accuracy. Recently, there has been a lot of research into lightweight vehicle semantic segmentation models. ESPNet [140] employs efficient convolutional modules, which are 22 times faster and 180 times smaller than existing state-of-the-art vehicle semantic segmentation networks. DFANet [141] begins with a solitary lightweight backbone and progressively consolidates discriminative features through a cascade of sub-networks and sub-stages. Experiments show that the model attained 1.7 GFLOPs at a speed of 160 FPS on one NVIDIA Titan X GPU and a 0.703 mIoU (mean IoU) on the Cityscapes dataset. LEDNet [142] utilizes an asymmetric encoder–decoder architecture and achieved 71 FPS and a 0.706 mIoU on the Cityscapes dataset with NVIDIA Titan X. Lightweight deployment capabilities will be a key technology for researchers to consider in the field of autonomous driving.

## 4. Vehicle Detection Algorithms Based on Radar and LiDAR

Vehicle detection using radar and LiDAR is a key component of modern advanced driver assistance systems (ADASs) and self-driving vehicles. LiDAR and radar differ from the visible light images captured by cameras that they acquire information about the distance and shape of the target. Both are now widely used in autonomous driving systems for intelligent vehicles.

### 4.1. Millimeter-Wave Radar-Based Methods for Vehicle Detection

**Figure 3:**
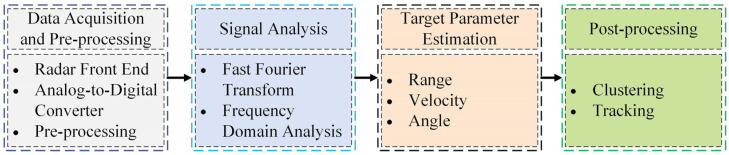
The radar-based vehicle detection process.

**Figure 4:**
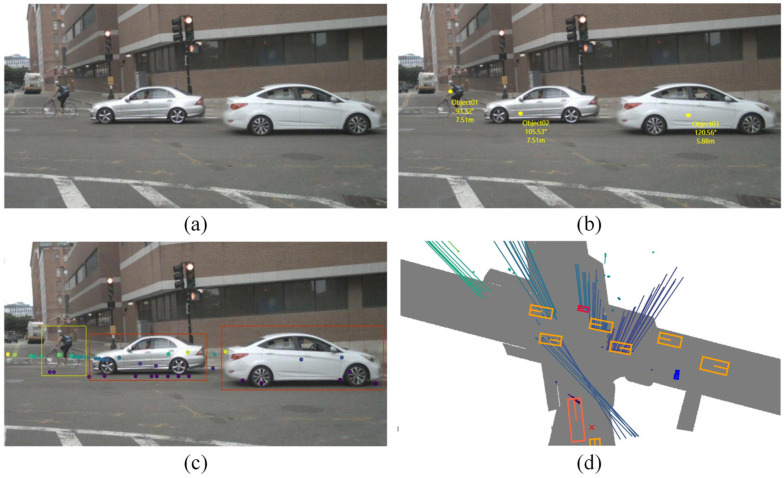
An example of target-level radar and image-level radar for vehicle detection: (**a**) original image, (**b**) target detection results (target-level), (**c**) projection map (image-level), (**d**) point cloud map (image-level) [27].

The millimeter-wave radar sensor operates by utilizing millimeter-wave frequencies in wireless radio wave detection. Its principle lies in the emission and reception of millimeter-wave signals, extracting parameters such as distance, velocity, direction, size, and trajectory of objects through techniques like time-of-flight measurements and Doppler effects. Compared to camera-based and LiDAR-based sensors, millimeter-wave radar is more resilient and allows vehicle detection in harsher weather conditions. In addition, these radars can also acquire accurate vehicle depth information, thus facilitating the perception of autonomous driving. The radar-based vehicle detection process is shown in Figure 3. Depending on the type of output signal, millimeter-wave radar is generally categorized into target-level radar and image-level radar. Figure 4 presents an example of this.

#### 4.1.1. Target-Level Radar

The target-level radar is oriented to the output target and can transform received echo signals directly into target information, such as distance, speed, and angle of the detected vehicles. Radar detection results can be classified into three categories: moving targets, stationary targets, and false targets. Dynamic vehicles, bicyclists, and pedestrians are the most common moving targets, while stationary targets mainly include parked automobiles, streetlamps, roadside trees, road guardrails, and curbs. False targets are invalid owing to interference or background noise. The radar itself does not have the discriminatory ability to classify detected targets. Therefore, there is a need to eliminate the interference of stationary and false targets on vehicle detection as much as possible. According to research, false target signals only remain for a short time when detected and can be eliminated by the Kalman filter [143], multiple-hypothesis target [144], and iterative adaptive approach [145]. In addition, some scholars have found that the radar cross-section (RCS) and signal-to-noise ratio (SNR) of stationary vehicles are much smaller than those of moving vehicles [146]. RCS refers to the extent to which an object is detected by radar, while SNR is the ratio of the desired signal power to the noise power. According to the characteristics of the motion state, a specific threshold value is set for the RCS and SNR of the radar, which can separate the moving vehicle targets from the stationary ones. Recognition and classification can be achieved by an ML-based classifier such as SVM and deep belief network (DBN).

The target-level radar provides information regarding the vehicle’s position and motion status, which is crucial for the environmental perception of autonomous driving. However, it lacks the ability to depict the vehicle’s contour and type. Furthermore, the detection accuracy of radar is not satisfactory when the vehicle is moving slowly or stationary. Hence, depending solely on target-level radar as a vehicle sensor is inappropriate.

#### 4.1.2. Image-Level Radar

Image-level radar is increasingly being applied in autonomous driving due to the need for high-resolution imaging. It not only provides information on the speed and motion status of the target but also generates an imaging map of the radar signals. In general, radar image formats can be categorized into four types: projection maps, range–Doppler–azimuth maps, point cloud maps, and SAR maps. By projecting the reflection intensity of a radar detection target onto the image, the reflection intensity map can be produced [12].

The generation of range–Doppler–azimuth maps requires the use of Fourier transform and time–frequency domain analysis techniques combined with distance measurements, Doppler shifts, and azimuth estimation algorithms. Both projection maps and range–Doppler–azimuth maps are 2D imaging maps that can be represented with deep learning algorithms, such as CNN [147], FCN [148], and LSTM [149].

Point cloud maps represent spatial data composed of a collection of points in the 3D coordinate system. Machine learning and deep learning algorithms are often used to model point cloud information for vehicle classification and detection. It has been reported that the radar point cloud can be clustered together using the Density-Based Spatial Clustering of Applications with Noise (DBSCAN) algorithm, allowing the obtained clustered vectors to describe the vehicle features [150]. The vehicle targets were then classified by SVM. However, the detection performance of millimeter-wave radar is limited by its low resolution. To improve this problem, a GAN architecture has been designed for recovering high-frequency shapes from original low-resolution millimeter-wave heat-maps [151]. According to this research, a CNN-based point cloud segmentation algorithm is utilized to detect vehicle targets, which can accurately reconstruct cars in real scenes with low visibility. Li et al. [152] developed a method to enhance radar perception with temporal information. They used the temporal relational layers of successive ego-centric bird-eye-view radar image frames for radar object recognition. Synthetic aperture radar (SAR) is a technique that produces fine-resolution coherent images from a resolution-limited radar system. SAR obtains image data by processing the reflected echoes, which can be used for vehicle detection with deep learning algorithms, such as CNN [153] and YOLO [154].

Millimeter-wave radar has been widely used in the field of autonomous driving for its robustness and anti-interference. With the continuous development of radar technology, how to further improve the resolution of radar will become a key research direction in the future.

### 4.2. LiDAR-Based Methods for Vehicle Detection

LiDAR is an optical technology that senses distance by measuring the time lapse between an emitted laser pulse and the detection of a reflected light pulse. In the process, LiDAR feeds back the geometric information about the object, such as size and 3D coordinates. The point cloud, composed of a collection of 3D points, can express the sensory information of the transportation environment. Compared with cameras and millimeter-wave radar, LiDAR has higher detection accuracy and can more accurately acquire information about the surroundings of the vehicle [4]. Moreover, it is insensitive to changes in light intensity, making it more applicable to vehicle detection in autonomous driving. LiDAR-based vehicle detection methods can be divided into two categories: traditional and deep learning.

#### 4.2.1. Traditional-Based Methods

**Figure 5:**
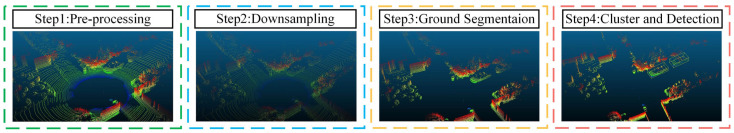
Traditional-based vehicle detection process using LiDAR point cloud.

Traditional methods rely on the construction of feature engineering and data processing. The traditional LiDAR vehicle detection algorithm is shown in Figure 5. For vehicle detection in a single frame point cloud, the raw image needs to be pre-processed, downsampled, ground-segmented, and clustered for feature extraction, respectively. Due to the sparse nature of LiDAR point cloud data, it is often necessary to convert 3D LiDAR data to 2D or 2.5D data to improve computational efficiency. These conversion techniques include a graph method [155], range image [156], and occupancy map [157]. Some irrelevant point cloud information can be eliminated to optimize the traffic environment sensing system. Studies have shown that the point cloud information of road pavement is significantly different from vehicles and other obstacles. It has been reported that road point clouds can be eliminated by setting specific feature thresholds to reduce the amount of computation and improve detection in real time [158,159]. However, these methods fail to perform well when dealing with special road sections, such as potholes or steep slopes. In this regard, scholars have proposed some fitting algorithms to solve the problem by partitioning the uneven pavement into a combination of several smooth planes, such as Markov random fields (MRFs) [160], random sampling consensus (RANSAC) [161], and Gaussian process regression (GPR) [162]. Next, the point cloud information with the same features is grouped with a clustering algorithm to highlight the attributes of the target. DBSCAN and K-means are classical clustering algorithms that divide points into clusters by density and distance, respectively. The clustered results are generally fed into the machine learning-based classifier, and then the vehicle detection is performed.

Traditional methods are simple to implement but are overly dependent on a priori knowledge in processing point cloud information. In addition, the process of vehicle detection requires multiple steps and cannot meet the real-time requirements in real scenarios.

#### 4.2.2. Deep Learning-Based Methods

**Figure 6:**
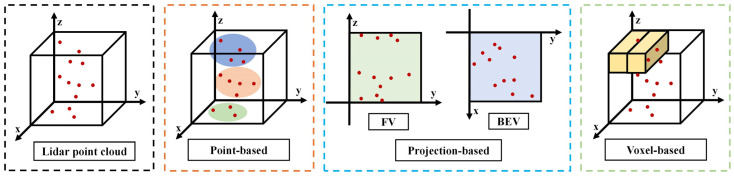
Spatial data representation of LiDAR point cloud.

With the rapid development of computer vision, LiDAR deep learning-based algorithms for vehicle detection demonstrate superiority in detection speed and accuracy. Most of these methods adopt an end-to-end approach, which facilitates the improvement in real-time vehicle detection. In contrast to the construction of feature engineering, deep learning algorithms can automatically learn complex point cloud information and extract high-level representation from deep networks. Based on the principle of the algorithms, LiDAR deep learning-based methods can be classified as point-based, projection-based, and voxel-based. The visual representations of these methods are shown in Figure 6.

(1) Point-Based Methods

Point-based methods perform 3D detection techniques of raw point cloud data to obtain vehicle targets. The primary characteristic of point cloud data is their insensitivity to the arrangement order of points. This implies that we can process point cloud data in any order. Vote3deep [163] employs a feature-centric voting scheme for constructing convolutional layers, which leverage the sparsity inherent in point cloud data. PointNet [164] is a classical algorithm presented in 2017. This method designs a novel neural network that processes point cloud information directly while respecting the permutation invariance of the input points. However, the design of PointNet fails to capture the local structure created by metric space points, restricting its ability to gather fine-grained information. PointNet++ [165] improves on PointNet to fully extract global and local vehicle features. It utilizes the PointNet network recursively on the set of input points through a hierarchical approach and adaptively combines features from multiple scales at the learning layer. PointRCNN [166] references the feature point extraction method of PointNet++ and utilizes a two-stage framework for detection and segmentation. Stage one generates 3D proposals through a bottom–up approach, and in the second stage, the convergence points of each proposal are converted to canonical coordinates. These models have excellent detection accuracy but are time-consuming. To achieve a reasonable balance between accuracy and efficiency, scholars propose a single-stage anchor-free-based detection method named 3DSSD [167]. This method adopts a fusion sampling strategy in downsampling to enable detection on fewer representative points, which yields the inference speed of 25+ FPS.

Point-based methods maximize the use of raw information from point clouds in space, which is effective for vehicle detection. However, the target is usually represented by only some of the points, resulting in a loss of spatial information between neighboring localized ones.

(2) Projection-Based Methods

Projection-based methods project a 3D point cloud onto a 2D plane to create a front view (FV) or bird’s eye view (BEV), which reduces the complexity of modeling point cloud data and requires fewer computational resources. DeepthCN [168] proposes a vehicle detection system based on hypothesis generation and hypothesis verification. The data input to the system is first subjected to ground segmentation and point cloud segmentation, and then it is projected onto a Dense-depth Map and detected by ConvNet. RT3D [169] projects the 3D point cloud onto the BEV and applies R-FCN for feature map extraction. BirdNet [170] projects laser information into a novel cell encoding for BEV, and then employs a CNN-based network to estimate the location and heading of the object, which is mapped through post-processing to 3D orientation detection. BirdNet+ [171] discards the post-processing step of BirdNet and achieves state-of-the-art results through an end-to-end detection framework for direct inference of oriented 3D boxes in BEV images.

LiDAR is expensive but is widely used as it can acquire 3D information of the environment with high accuracy. In projection-based methods, the feature map is eventually projected as a 2D map, which is similar to 2D target detection. Consequently, some scholars have proposed pseudo-LiDAR representations, which essentially mimic LiDAR signals. Wang et al. [172] converted depth maps to pseudo-LiDAR maps and improved the detection accuracy of within 30 m to 74% in the KITTI dataset. However, pseudo-LiDAR-based methods generally require the depth estimation of the 2D map before performing 3D object detection, resulting in two separate steps. Qian et al. [173] introduced a framework based on the differentiable change in the representation module that allows end-to-end training of the entire pseudo-LiDAR pipeline. Pseudo-L [174] presents three novel methods for virtual view generation, including image-level generation methods, feature-level generation, and a feature clone. Furthermore, a disparity-wise dynamic convolution is proposed, which alleviates the feature degradation caused by depth estimation errors.

The success of projection-based methods is essentially the maturation of 2D detection algorithms. However, dimensionality reduction in the 3D point cloud information will inevitably result in the loss of spatial depth information, reducing the detection accuracy.

(3) Voxel-Based Methods

The point cloud data collected by LiDAR are typically dense, yet a majority of points are concentrated within specific spatial regions. This leads to the sparsity of data, making direct processing of the entire point cloud complex and inefficient. In order to better describe the distribution of the point clouds in three dimensions, the challenge is solved by dividing the point clouds into regular grids of voxels of a specific size. VoxelNet [175] is a representative algorithm that combines PointNet and CNN to present an end-to-end trainable architecture. The model represents voxel points through a voxel feature encoding (VFE) layer, which enables efficient parallel processing of the voxel grid. Voxel RCNN [176] designs a voxel ROI pooling to further refine the features of the BEV region proposal network. PV-RCNN [177] summarizes the 3D scene with 3D voxel CNNs into a small collection of keypoints and then employs ROI grid points to extract richer contextual information. PV-RCNN++ [178] proposes two improvements based on PV-RCNN, sectorized proposal-centric sampling and vector pool aggregation, which can generate more efficient keypoints and better aggregate local point features, respectively. VoxelNeXt [179] directly uses a sparse convolutional network to detect and track 3D objects entirely through voxel features without switching to a dense detection header and NMS post-processing, resulting in a better trade-off between speed and accuracy. Other voxel-based methods include MA-MFFC [180], PDV [181], and SAT-GCN [182].

The voxel-based approach converts the point cloud data into a 3D voxel grid, which offers the advantages of high processing efficiency and good spatial information retention. Nevertheless, some information might be lost using this method. Table 4 summarizes the performance of different LiDAR-based deep learning models.

#### 4.2.3. Point Cloud Segmentation-Based Methods

In the context of road scenes, semantic segmentation labels each point to a predefined category, such as pedestrians, vehicles, trees, etc. Vehicle detection algorithms based on point cloud segmentation can be divided into two categories: traditional methods and deep learning-based methods. Traditional segmentation approaches rely on prior knowledge and feature engineering, like region growing [199], clustering [200], and model fitting [201]. The design of feature engineering entails substantial time investment, while the determination of segmentation boundaries through thresholding is prone to errors. Moreover, achieving pixel-level segmentation poses significant challenges. These factors collectively contribute to the limitations of traditional methods in point-cloud-based vehicle detection. Presently, deep learning-based point cloud segmentation has achieved remarkable performance in accuracy and speed. These approaches are classified as point-based, projection-based, and voxel-based.

(1) Point-Based Methods

These methods directly process 3D point cloud information for vehicle detection. PointNet [164] and PointNet++ [165] are the most representative models in this domain. They leverage shared Multi-Layer Perceptrons (shared MLPs) and pooling to integrate global and local features, while employing MLPs to assign semantic labels to individual points. However, these point sampling methods exhibit poor scalability with respect to the scale of point clouds. Additionally, employing max-pooling to group local points may result in robustness in complex scenes. Some scholars have attempted to address these issues. RandLA-Net [202] integrates random point sampling with a local feature aggregation module to increase the receptive field of each 3D point, rendering it suitable for the semantic segmentation of large-scale point clouds on a per-point basis. S3Net [203] utilizes sparse mechanisms to construct modules, thereby providing rich contextual information for feature maps. Direct processing of point clouds can also be achieved through point convolution. KPConv [204] identifies a set of pivotal points in the spatial domain, employing kernel functions to compute the weighting coefficients for each point, thereby effecting a transformation of the features. Landrieu et al. [205] proposes a framework for a large-scale point cloud based on the concept of a superpoint graph (SPG). The SPG facilitates the provision of compact yet rich contextual information, which can increase the performance of point cloud segmentation.

(2) Projection-Based Methods

The sparsity and lack of structure in point clouds pose challenges for feature extraction using CNN in 3D space. Projection-based point cloud segmentation converts 3D point clouds into 2D BEV maps, FV maps, and RV maps and then uses CNN for feature extraction, followed by reconstructing the original 3D scenes. SqueezeSeg [206] employs spherical projection to transform point clouds into front-view representations. Then, SqueeNet [207] is used to output a point-wise label map, which is refined by a conditional random field. SqueezeSegv2 [208] introduces a context aggregation module to enhance SqueezeNet and proposes an adaptive training approach to reduce the distribution gap between simulated data and real data. SqueezeSegv3 [209] introduces spatially adaptive convolution, which employs different filters for various positions in the image. In order to minimize the loss of information due to dimensional changes during projection, some scholars have proposed multi-view projection methods. GVCNN [210] is a typical representative algorithm. It groups feature subgraphs from different viewpoints based on discriminative weight and then aggregates descriptions of each group through pooling.

(3) Voxel-Based Methods

The sparsity and lack of structure of point clouds greatly affects their representation capability. Voxel-based point cloud segmentation transforms point clouds into structured voxels and employs 3D networks for semantic segmentation. In this process, the depth information is fully utilized at the expense of resolution. VoxNet [211] is a pioneer in this approach, utilizing 3D CNN to process the voxels of the occupied girds. Subsequently, a number of voxel-based algorithms have been applied to vehicle detection, such as SegCloud [212], Kd-Net [213], and SPVNAS [214]. These models require a lot of computational resources and memory space, which is detrimental to real-time 3D segmentation. PointGrid [215] is a hybrid model that integrates a point and grid, using simple points to quantify local features for each grid unit. Further, considering the geometric spatial properties of 3D point clouds, some scholars have attempted to optimize the network structure using octrees, such as OctNet [216] and O-CNN [217]. Recently, Hou et al. [218] proposed applying knowledge distillation to the semantic segmentation of LiDAR for model compression. The method suggests inter-point and inter-pixel affinity distillation and uses a difficulty-aware sampling strategy for difficult hypervoxels.

**Figure 7:**
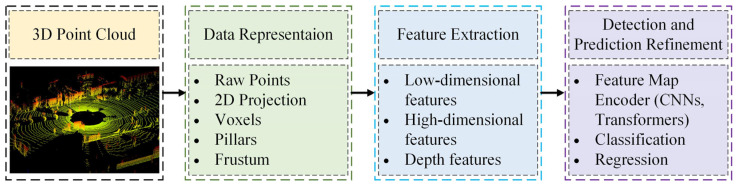
Pipeline for vehicle detection based on LiDAR deep models [15].

Projection-based methods and voxel-based methods lead to the loss of point cloud information. In contrast, point-based methods effectively retain the original point cloud data but incur higher computational costs. In recent years, point cloud segmentation methods have emerged as a significant research focus for LiDAR-based vehicle detection. Some of these algorithms are not specifically designed for vehicle detection but serve as general network architectures that can be adapted to this domain through retraining. Overall, the deep learning-based and the point cloud segmentation-based approach to achieve vehicle detection can be summarized as the process shown in Figure 7.

## 5. Vehicle Detection Algorithms Based on Multi-Sensor Fusion

Section 3 and Section 4 summarize the vehicle detection algorithms under three typical sensors: a camera, millimeter-wave radar, and LiDAR. It can be noted that each sensor has its advantages and limitations related to its functional operation. A number of metrics for these sensors are summarized in detail in Table 5. In autonomous driving, environmental perception presents a multifaceted challenge. The integration of multiple sensors facilitates synergistic advantages, leading to augmented information acquisition. In general, multi-sensor fusion can be categorized into stereo vision-based, fusion of millimeter-wave radar and vision-based, fusion of LiDAR and vision-based, and multi-sensor-based.

### 5.1. Stereo Vision-Based Methods for Vehicle Detection

**Figure 8:**
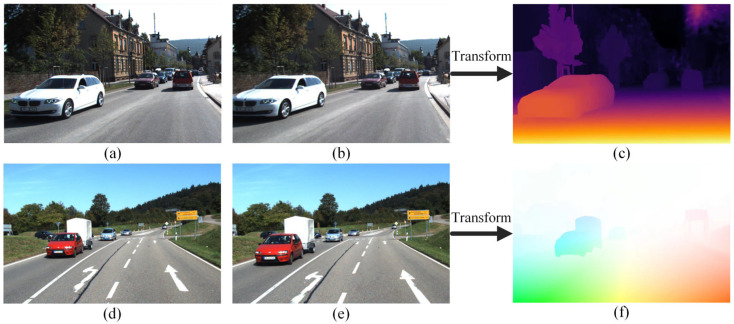
An example of appearance-based methods and motion-based methods for vehicle detection: (**a**) left-eye view, (**b**) right-eye view, (**c**) disparity map (appearance-based); (**d**) a frame from the video stream, (**e**) the subsequent frame from the video stream, (**f**) the optical flow map (motion-based) [15].

Stereo vision is a technique that utilizes two or more cameras to simultaneously capture images of a scene from different perspectives in order to obtain depth information and position information of a target in space. Based on the idea of estimating a target’s parameters through the changes in the corresponding points in the disparity map, this technique draws inspiration from the search mechanism of the human eyes. According to the principle of the algorithms, stereo vision techniques can be classified into appearance-based methods and motion-based methods, shown as Figure 8.

(1) Appearance-Based Methods

These methods rely on the extraction of salient vehicle appearance features to achieve vehicle detection. The U-V disparity is a common method used in vehicle detection [219]. Xie et al. [220] proposed a stereo vision segmentation algorithm based on a cascaded framework. For a corrected binocular image pair disparity map, a probabilistic approach is used to compute the U-V disparity, and outliers are removed using RANSAC to obtain the road region. Ma et al. [221] used a nonparametric and refined U-V disparity mapping method to obtain the road ROI, and then utilized an adjacent disparity similarity algorithm to complement and extract the target region for vehicle detection. Observing that the depth information of vehicles is constantly changing in stereo vision, some scholars used depth information clustering to detect vehicles [222]. In recent years, researchers have reported methods for vehicle environmental perception using machine learning and deep learning, which have achieved satisfactory detection results [223,224].

(2) Motion-Based Methods

This type of algorithm relies heavily on the optical flow information. Optical flow information refers to the displacement of pixel points between consecutive image frames due to the motion of an object. Kale et al. [225] make use of optical flow in conjunction with motion vector estimation for object detection and tracking in a sequence of frames. Sengar et al. [226] utilized a Gaussian filter to remove noise from each frame and detected moving targets by calculating the optical flow between three consecutive frames. Some scholars have proposed the fusion of vehicle depth information with optical flow information through occupancy grids to strengthen representation capability and further enhance detection efficiency. Chen et al. [227] optimized the classical optical flow algorithm at a single resolution on a regular grid. Yin et al. [228] presented GeoNet, a joint unsupervised learning framework. This method combines depth, optical flow, and self-motion estimation for image reconstruction loss, and inference for static and dynamic scene parts.

### 5.2. Fusion of Radar and Vision-Based Methods for Vehicle Detection

**Figure 9:**
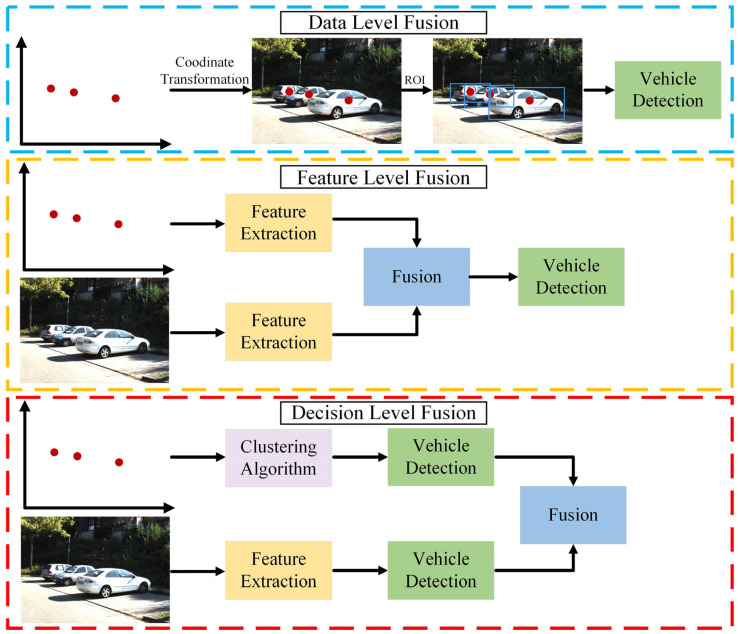
Different methods of radar–camera fusion for vehicle detection [15].

Millimeter-wave radar exhibits strong adaptability to complex environments and offers motion and depth information about vehicles. With their high-resolution imaging capabilities, cameras excel in detection and classification tasks. The integration of these two sensors is a typical configuration in many mature autonomous driving systems [14]. Figure 9 illustrates the three fusion levels that are often present for radar and vision-based methods, including the data level, decision level, and feature level.

(1) Data-Level Fusion

Data-level fusion is a well-established method for vehicle detection by fusing camera data and radar data. Although this scheme is not currently a mainstream method, its fusion idea is worthwhile. Specifically, data-level fusion first generates ROI through radar, then visual images analyze these regions, and finally vehicle targets are obtained through detectors. Wang et al. [229] employed a fusion strategy of visual attention mechanisms to detect vehicles via an adaptive thresholding algorithm. Wang et al. [230] optimized the fusion approach to achieve the balance between vehicle detection accuracy and speed. Craft [231] focuses on the spatial properties of the camera and radar, adaptively fusing spatial contextual information between the two.

(2) Feature-Level Fusion

Feature-level fusion is a processing approach for features extracted from raw data. This type of fusion is applicable to a collection of features extracted from multiple sensors or data sources. For the feature extraction of radar information, radar points can be converted into image format. Then, for the feature maps obtained from each sensor, deep models (e.g., CNNs and Transformers) can be used to achieve feature fusion. Lekic et al. [232] introduced a conditional multi-generator generative adversarial network. The model can qualitatively and quantitatively convert environmental features detected by radar sensors into visually appealing images. Chang et al. [233] presented a novel spatial attention fusion method for vehicle detection. Starting with the sensor features, the method fuses the vision features by applying an attention weight matrix. Zhou et al. [234] contributed to multimodal fusion 3D object detection by narrowing the view disparity in different sensor features.

(3) Decision-Level Fusion

Decision-level fusion is the highest level of image fusion in which the independent detection results from each of the camera and radar sensors are integrated. The advantage of radar lies in its ability to accurately measure the longitudinal distance of a target, while the camera provides a broader field of view. Combining radar and a camera can fully utilize these two types of information, thereby improving the accuracy and reliability of target detection and tracking. Zhong et al. [235] reported a Kalman-filter-based camera–radar fusion system, which strikes a balance between performance and energy efficiency and demonstrates the competitiveness of the software–hardware ecosystem. Bai et al. [236] correlated the respective detections of the radar and camera in the image plane to generate a random finite set with an object type. The model is then refined using a Gaussian mixture probability hypothesis density algorithm. Sengupta et al. [237] combined the Hungarian algorithm and triple Kalman filtering for object tracking, significantly reducing the false-negative rate and providing a promising direction for autonomous perception. Table 6 summarizes the performance of some of the latest different radar–camera-based deep learning models.

### 5.3. Fusion of LiDAR and Vision-Based Methods for Vehicle Detection

**Figure 10:**
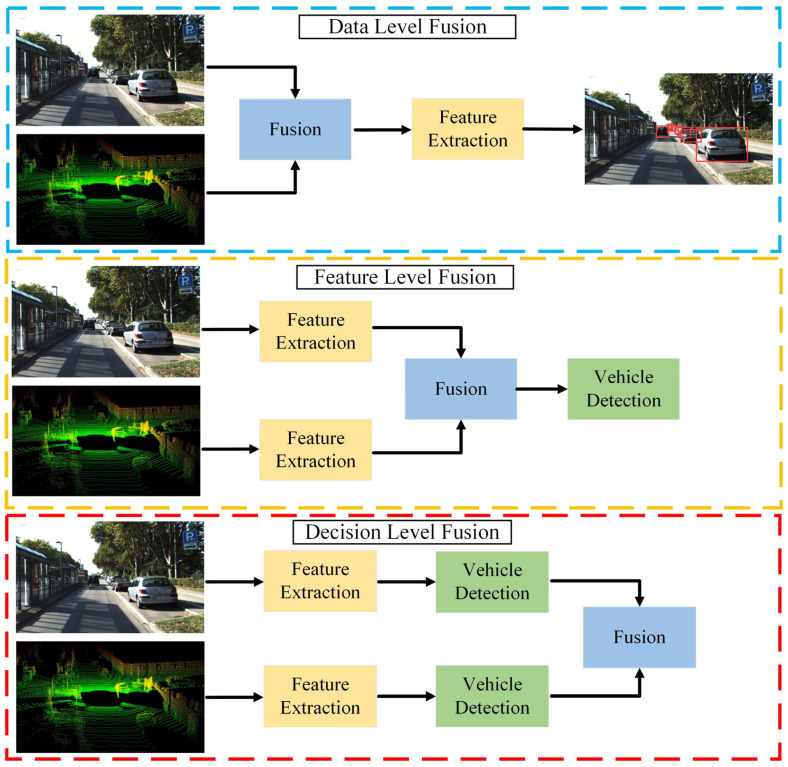
Different methods of LiDAR–camera fusion for vehicle detection [15].

Compared to millimeter-wave radar, LiDAR possesses a superior manufacturing process, which allows it to deliver higher precision and resolution in imaging technology. As a result, the integration of these two sensors is considered to be an outstanding method for environmental perception. Figure 10 illustrates the three fusion levels that are often present for LiDAR and vision-based methods, including the data level, decision level, and feature level.

(1) Data-Level Fusion

The raw data captured by LiDAR and a camera are inherently different in structure, which renders data-level fusion complex and potentially detrimental to the quality of information representation. As a result, this approach is currently no longer universally applicable in the context of environmental perception.

(2) Feature-Level Fusion

This approach integrates data extracted from LiDAR and a camera through a feature extraction mechanism. MV3D is a typical feature-level fusion framework [241]. This model takes the FV and BEV of the LiDAR, along with an image from a camera, as inputs, and projects the 3D proposals generated in the BEV into three views. Then, a feature fusion network is employed to fuse the feature maps obtained from ROI pooling of each view for vehicle detection. Ku et al. [242] used RPN to achieve multimodal feature fusion on high-resolution feature maps, followed by 3D regression and classification. Ku et al. proposed a method to accurately estimate the 3D bounding box [243]. The scheme first employs a 2D detector, and then lifts the 2D region to 3D to generate stymied proposals for 3D bounding box estimation. Zhao et al. [244] used LiDAR data to generate region proposals, and fed the generated ROIs from candidates into a CNN for vehicle detection. An et al. [245] leveraged the geometric consistency between 3D and 2D local regions, integrating manually crafted 2D features with attention-based voxel features to enhance the accuracy of 3D object detection. Li et al. [246] initially employed residual modules to extract image features and sparse convolutions to extract BEV map features from a LiDAR point cloud. Subsequently, these features were fused and fed into the RPN to detect vehicles. Liang et al. [247] converted multi-view images and LiDAR point clouds into BEV maps separately, and then used a dynamic fusion module to fuse the two feature maps for 3D target detection, which overcame the limitation of over-reliance on LiDAR data. Liu et al. [248] constructed a shared BEV map, thus preserving the semantic density of the camera and the geometry of the LiDAR. In addition, it can be extended to multi-task, multi-sensor frameworks. Wu et al. [249] proposed VirConvNet, a fast and efficient virtual point-based 3D object detection backbone network based on the VirConv operator, which reduces redundant computation and noise interference. The application of feature-level fusion improves the representation capability and thus helps to improve the accuracy of vehicle detection. However, the use of multiple feature extraction modules to obtain feature maps from sensors may lead to a decrease in speed.

(3) Decision-Level Fusion

A fusion algorithm is applied to the objects detected by the camera or LiDAR, respectively, in decision-level fusion. Typical fusion algorithms include the Kalman filter, Bayesian, etc. Oh et al. [250] used a CNN to fuse the classification outputs of an independent unary classifier. This classifier utilizes more than two pre-trained convolutional layers to consider local-to-global features as data representations. Guan et al. [251] proposed a decision-level object detection method based on Dempster–Shafer evidence theory. They complemented and converted the 2D LiDAR sparse depth map to a dense depth map, then used YOLOv3 [85] for vehicle target detection on both an RGB image and depth map, and then fused the two results to receive the information of vehicles. The decision-level approach has excellent robustness and ensures the normal operation of the system even if one of the sensors fails. At present, there is less research on decision-level methods compared to feature-level methods. Table 7 summarizes the performance of different LiDAR–camera-based deep learning models.

### 5.4. Multi-Sensor-Based Methods for Vehicle Detection

The original intentions behind the design of each sensor are different, and the fusion of multiple sensors to achieve multimodal perception is a promising trend for vehicle detection in autonomous driving in the future. Chavez-Garcia et al. [271] proposed a complete perception fusion architecture based on an evidence framework that combines composite representation with uncertainty management to solve the detection and tracking problem of moving targets. Yi et al. [272] presented a spatial calibration algorithm based on a multi-sensor system. They fused LiDAR, radar, and a camera to detect and recognize targets. In the realm of autonomous driving, environmental perception and vehicle detection typically entail the aggregation of information from disparate sensors. Therefore, multi-sensor fusion solutions can also be extended to encompass research on the fusion of one or two sensors.

## 6. Discussion and Future Trends

Section 3, Section 4 and Section 5 of this paper provide a detailed overview of the mainstream self-driving vehicle detection algorithms. In this section, we will discuss the algorithms for different sensors and explore the future trends of vehicle detectors.

### 6.1. Discussion

(1) Machine Vision

Camera sensors typically use stereo vision technology to acquire depth information of objects by comparing the disparity between images captured by two cameras to calculate the distance of vehicles from the cameras. Once the camera sensor captures the image, vehicle detection algorithms such as CNNs are employed to identify vehicles in the image and determine their positions and bounding boxes. The bounding boxes provide information about the size and orientation of the vehicles. By analyzing images from consecutive frames to recognize the displacement of objects, their speed and direction can thereby be determined. In the realm of machine-vision-based vehicle detection, deep learning approaches have taken the lead. Due to the powerful fitting and representation capabilities of deep models, they can extract deeper feature information, and thus are proven to be the optimal choice for vehicle detection. The vehicle detection algorithm based on semantic segmentation possesses a finer-grained representation compared to object-detection-based methods, and achieves higher precision. However, the trade-off in detection speed is a matter worth considering. The research on vehicle detection algorithms for camera sensors presents a diverse landscape, with each method having advantages and disadvantages. However, given the critical importance of speed metrics in autonomous driving, the design of algorithms must prioritize real-time performance.

(2) Millimeter-Wave Radar

Millimeter-wave radar can transmit millimeter-scale electromagnetic pulse wave energy and analyze the echo signal to receive the position and motion status of the vehicles. By utilizing antenna array processing, the millimeter-wave radar can derive angular data from a vehicle’s reflection points. Upon encountering an object, the emitted millimeter-wave signal undergoes partial absorption and reflection, with the reflected signal returning to the sensor. By measuring the time difference between the transmitted and received signals, the distance between the object and the sensor is determined. This allows these points to be located in 3D space when combined with the time of flight. Millimeter-wave radar, as an inexpensive sensor, is capable of operating in all-weather conditions. However, millimeter-wave radar has low resolution and cannot provide information on the type and size of vehicles, which are indispensable requirements for vehicle detection. Therefore, millimeter-wave radar is usually used as an auxiliary sensor or in combination with other sensors. Nevertheless, its adaptability to complex weather conditions has led to its widespread application in the field of autonomous driving.

(3) LiDAR

LiDAR is an active sensor that plays a crucial role in detecting vehicles for autonomous driving due to its high precision and optical stability. The operation principle of a LiDAR sensor includes a laser emission, reception, and analysis. Firstly, a short-pulse laser beam is generated by the laser emitter to record its round-trip time, resulting in distance information. Subsequently, through the rotation or scanning of the sensor, LiDAR can acquire reflected signals in different directions, thus constructing a point cloud in three-dimensional space to achieve vehicle localization. By identifying and extracting parameters from the point cloud information, the size and shape of vehicles can be obtained. Methods based on deep learning and point cloud segmentation have been widely applied in vehicular radar systems. However, the sparse and unstructured nature of a point cloud leads to high computational costs when processing raw data directly. Researchers have adopted various data representation methods to structure the original point cloud and then utilize deep models for vehicle detection. This approach sacrifices some point cloud information in exchange for higher detection efficiency. LiDAR can function as an independent sensor or be fused with cameras for perception, representing a classic environmental perception scheme.

(4) Sensor Settings

Table 8 shows the sensor solutions of some autonomous driving manufacturers. We can see that Tesla and Xpeng have opted for a combination of cameras, millimeter-wave radar, and ultrasonic radar, rather than using LiDAR as their fundamental perception sensor. Other manufacturers have opted for LiDAR sensors, with Waymo in particular using four LiDAR sensors. It can be concluded that the mainstream trend in the field of environmental perception for autonomous vehicles is the fusion of radar and a vision sensing solution. Radars and cameras possess complementary characteristics, allowing for better perception in real-world environments.

### 6.2. Future Trends

(1) Balancing Speed and Accuracy of Algorithms

The performance of vehicle detection algorithms directly influences the perception capability of autonomous driving systems. In this field, the speed and accuracy of algorithms are the core issues in environmental perception for intelligent vehicles. Many algorithms exhibit either high precision or fast speed in practical deployment, but few can simultaneously achieve both aspects. Some scholars attempt to enhance one aspect of performance at the expense of another, which often lacks robustness in real driving scenarios. The performance of deep models depends on multiple prerequisites, with the design of the backbone network being considered one of the most critical factors. For general vehicle-embedded chips, lower computational complexity and faster processing speed are prioritized. Hence, exploring the effectiveness of network architectures while ensuring low algorithmic complexity is an intriguing research direction. One of the future research focuses will be on designing superior backbone network architectures to achieve a balance between accuracy and speed.

(2) Multi-Sensor Fusion Strategy

Vehicle detection algorithms based on machine vision, millimeter-wave radar, or LiDAR each possess distinct advantages and drawbacks. Inherent limitations persist in vehicle detection algorithms reliant on a singular sensor modality, rendering them unavoidably constrained. Therefore, multi-sensor fusion to realize cooperative perception will be a hot research topic in the future. Currently, industrial-grade autonomous driving is typically deployed with a multi-sensor fusion strategy, which is proven to be effective. Nonetheless, fusion techniques for vehicle detection still encounter challenges such as immaturity and representation disparities. To enhance the representation capabilities of multi-sensor fusion, further consideration in design schemes and protocols is necessary. For algorithms, research on enhanced fusion algorithms is imperative to maximize the utilization of non-redundant multiscale information for a collaborative perception of multiple sensors.

(3) Multi-tasking Algorithms

Existing vehicle detection methods, such as target detection and semantic segmentation, are experimented in specific traffic scenarios. Different algorithms are often optimized for varying usage contexts. However, practical vehicle detection in real-world traffic environment scenes confronts challenges of diversity and complexity, including adverse weather conditions such as fog, night-time, snow, and rain. Presently, most algorithms are tailored to specific scenes, lacking a universal approach capable of adaptive detection across various environments. Therefore, coping with vehicle detection in complex environments becomes an inevitable trend in the future. In future research, the integration of diverse algorithms into a framework adaptable to dynamic traffic conditions should be pursued. This can not only enhance detection speed and accuracy but also, more importantly, augment the adaptability and robustness of the vehicle detection system, mitigating the occurrence of traffic accidents due to perception failures.

(4) Unsupervised Learning

Today, almost all mainstream vehicle detection algorithms are based on supervised learning. These methods require large amounts of well-labeled data to train the model, and tend to have outstanding performance in test sets. However, these models require large computational resources for dataset training, which is a time-consuming and laborious task. Additionally, models based on supervised learning exhibit certain limitations in terms of generalization; when confronted with scenes divergent from the training sets, the detection accuracy often suffers. Hence, one feasible direction for the future is the development of semi-supervised or weakly supervised vehicle detection algorithms to address this issue. These algorithms can make better use of unlabeled data and thus achieve more accurate vehicle detection across a broader range of scenarios.

## 7. Conclusions

Autonomous driving technology is gradually changing the way people commute and reshaping transportation systems. Vehicle detection, the capability to perceive vehicles in real driving scenarios, has long been a topic of great interest in the field of autonomous driving. In this paper, we have provided a comprehensive review of vehicle detection algorithms for autonomous driving. We started by introducing the tasks, evaluation metrics, and datasets. Second, a detailed analysis of various detection methods was presented, such as machine-vision-based, millimeter-wave-radar-based, LiDAR-based, and sensor-fusion-based approaches. Finally, we delved into various sensor modalities and their associated detection algorithms, emphasizing the crucial balance between precision, speed, and environmental adaptability, and provided an outlook on future research directions. The main contribution of this work is to summarize and analyze over 200 classical as well as state-of-the-art vehicle detection algorithms in an organized manner, which helps researchers to have a deeper and more comprehensive understanding of this field. In the future, more systematic and comprehensive perception techniques will become the research hotspots. Sensor fusion strategies, multi-task algorithms, and unsupervised learning methods show a very promising trend. At the same time, striking a more reasonable balance between detection speed and detection accuracy has posed a challenge for researchers. Capturing these key elements not only enhances vehicle detection efficiency but also promotes the development of autonomous driving technology.

## Figures and Tables

**Table 1:** Datasets for vehicle detection. Sc. stands for Scenes, Cl. for Classes, An. for Annotations, and 3Db. for 3D boxes.

Dataset	Year	Loc.	Sc.	Cl.	An.	3Db.	Application Scenarios
KITTI [15]	2012	Karlsruhe (DE)	22	8	15 k	200 k	Multiple application scenarios.
Cityscapes [16]	2016	50 cities	-	30	25 k	-	Mainly oriented to segmentation tasks.
Oxford RobotCar [17]	2016	Central Oxford (UK)	-	-	-	-	Multimodal joint calibration tasks can be conducted.
Vistas [18]	2017	Global	-	152	25 k	-	Globally constructed dataset for autonomous driving.
BDD100K [19]	2018	San Fransisco and New York (US)	100 k	10	100 k	-	The total volume of data is enormous, nearly 2 terabytes.
ApolloScape [20]	2018	4 cities in CN	-	8–35	144 k	70 k	It contains many extensive and richer labels.
KAIST [21]	2018	South Korea	-	3	8.9 k	-	Primarily targets SLAM tasks, emphasizing the provision of examples in complex scenarios.
Waymo open [22]	2019	6 cities in US	1 k	4	200 k	12 M	Focused on computer vision tasks, and utilizes data collected in all-weather conditions.
Lyft L5 [23]	2019	California (US)	366	-	-	55 k	More than 1000 h of driving record data.
Argoverse [24]	2019	Pittsburgh and Miami (US)	1 k	-	22 k	993 k	Focus on two tasks: 3D tracking and action prediction.
D^2^-City [25]	2019	5 cities in CN	1 k	12	700 k	-	Suitable for detection and tracking tasks.
H3D [26]	2019	San Francisco (US)	160	8	27 k	1.1 M	It is a large-scale full-surround 3D multi-object detection and tracking dataset.
nuScenes [27]	2019	Boston (US), Singapore	1 k	23	40 k	1.4 M	It was taken in dense traffic and highly challenging driving situations.
CADC [28]	2020	Waterloo (CA)	75	10	7 k	-	Focused on constructing a dataset for driving in snowy conditions.
A2D2 [29]	2020	3 cities in DE	-	14	12 k	43 k	Perception for autonomous driving.
A*3D [30]	2020	Singapore	-	7	39 k	230 k	With a significant diversity of the scene, time, and weather.
RADIATE [31]	2021	UK	7	8	-	-	Focus on tracking and scene understanding using radar sensors in adverse weather.
ACDC [32]	2021	Switzerland	4	19	4.6 k	-	A larger semantic segmentation dataset on adverse visual conditions.
KITTI-360 [33]	2022	Karlsruhe (DE)	-	37	-	68 k	An extension of the KITTI dataset. It established benchmarks for tasks relevant to mobile perception.
SHIFT [34]	2022	8 cities	-	23	2.5 M	2.5 M	A synthetic driving dataset for continuous multi-task domain adaptation.
Argoverse 2 [35]	2023	6 cities in US	250 k	30	-	-	The successor to the Argoverse 3D tracking dataset. It is the largest ever collection of LiDAR sensor data.
V2V4Real [36]	2023	Ohio (US)	-	5	20 k	240 k	The first large-scale real-world multimodal dataset for V2V perception.

**Table 2:** Different studies on feature engineering and classifiers for vehicle detection.

Feature	Classifier	Dataset	Accuracy	Reference
HOG	Adaboost	GTI vehicle database and real traffic scene videos	98.82%	[55]
HOG	GA-SVM	1648 vehicles and 1646 non-vehicles	97.76%	[56]
HOG	SVM	420 road images from real on-road driving tests	93.00%	[57]
HOG	SVM	GTI vehicle database and another 400 images from real traffic scenes	93.75%	[68]
Haar-like	Adaboost	Hand-labeled data of 10,000 positive and 15,000 negative examples	-	[69]
SURF	SVM	2846 vehicles from 29 vehicle makes and models	99.07%	[70]
PCA	SVM	1051 vehicle images and 1051 nonvehicle images	96.11%	[71]
SIFT	SVM	880 positive samples and 800 negative samples	-	[72]

**Table 3:** Comparison of detection performances on MS COCO dataset.

Model	Backbone	AP	AP_50_	AP_75_	AP_S_	AP_M_	AP_L_
Anchor-based two-stage							
Faster RCNN [73]	VGG-16	21.9	42.7	-	-	-	-
R-FCN [78]	ResNet-101	29.9	51.9	-	10.8	32.8	45.0
CoupleNet [110]	ResNet-101	34.4	54.8	37.2	13.4	38.1	50.8
Mask RCNN [76]	ResNeXt-101	39.8	62.3	43.4	22.1	43.2	51.2
DetNet [111]	DetNet-59	40.3	62.1	43.8	23.6	42.6	50.0
Soft-NMS [112]	ResNet-101	40.8	62.4	44.9	23.0	43.4	53.2
G-RMI [113]	-	41.6	61.9	45.4	23.9	43.5	54.9
Cascade R-CNN [80]	Res101-FPN	42.8	62.1	46.3	23.7	45.5	55.2
SNIP [114]	DPN-98	45.7	67.3	51.5	29.3	48.8	57.1
Anchor-based one-stage							
SSD [81]	VGG-16	28.8	48.5	30.3	10.9	31.8	43.5
DSSD [115]	ResNet-101	33.2	53.3	35.2	13.0	35.4	51.1
M2Det [82]	VGG-16	33.5	52.4	35.6	14.4	37.6	47.6
RefineDet [116]	ResNet-101	36.4	57.5	39.5	16.6	39.9	51.4
RetinaNet [83]	ResNet-101	39.1	59.1	42.3	21.8	42.7	50.2
YOLOv2 [84]	DarkNet-19	21.6	44.0	19.2	5.0	22.4	35.5
YOLOv3 [85]	DarkNet-53	33.0	57.9	34.4	18.3	35.4	41.9
YOLOv4 [86]	CSPDarkNet-53	41.2	62.8	44.3	20.4	44.4	56.0
YOLOv5 [87]	CSPDarkNet-53	49.0	67.3	-	-	-	-
YOLOv7 [88]	ELAN	52.9	71.1	57.5	36.9	57.7	68.6
Anchor-free keypoint-based							
CornerNet [90]	Hourglass-104	40.5	56.5	43.1	19.4	42.7	53.9
RepPoints [91]	Res101-DCN	45.9	66.1	49.0	26.6	48.6	57.2
CenterNet [92]	Hourglass-104	44.9	62.4	48.1	25.6	47.4	57.4
ExtremeNet [93]	Hourglass-104	40.2	55.5	43.2	20.4	43.2	53.1
Grid R-CNN [94]	ResNeXt-DCN	43.2	63.0	46.6	25.1	46.5	55.2
Anchor-free center-based							
FSAF [95]	ResNeXt-101	42.9	63.8	46.3	26.6	46.2	52.7
FCOS [96]	ResNeXt-101	43.2	62.8	46.6	26.5	46.2	53.3
GA-RPN [97]	ResNet-50	39.8	59.2	43.5	21.8	42.6	50.7
FoveaBox [98]	ResNeXt-101	42.1	61.9	45.2	24.9	46.8	55.6
YOLOX [99]	CSPDarkNet-53	50.0	68.5	54.5	29.8	54.5	64.4
YOLOv6 [117]	EfficientRep	52.8	70.3	57.7	34.4	58.1	70.1
YOLOv8 [100]	DarkNet-53	52.9	69.8	57.5	35.3	58.3	69.8
YOLOv9 [101]	GELAN	53.0	70.2	57.8	36.2	58.5	69.3
End-to-end-based							
DeFCN [102]	-	38.6	57.6	41.3	-	-	-
Sparse R-CNN [103]	ResNet-50	42.8	61.2	45.7	26.7	44.6	57.6
DETR [104]	ResNet-50	43.3	63.1	45.9	22.5	47.3	61.1
Deformable DETR [107]	ResNet-50	46.2	65.2	50.0	28.8	49.2	61.7
Anchor-DETR [108]	ResNet-101	45.1	65.7	48.8	25.8	49.4	61.6
Efficient-DETR [118]	ResNet-101	45.7	64.1	49.5	28.8	49.1	60.2
RT-DETR [109]	ResNet-101	54.3	72.7	58.6	36.0	58.8	72.1

**Table 4:** The performance of different LiDAR-based deep learning models on KITTI dataset.

Model	Car AP (IoU = 0.7)			FPS	Year	Reference
	Easy	Moderate	Hard			
Point-based						
Vote3deep	76.79	68.24	63.23	0.9	2017	[163]
PointRCNN	85.95	75.76	68.32	3.8	2019	[166]
STD	86.61	77.63	76.06	-	2019	[183]
Part-A2	85.94	77.95	72.00	-	2020	[184]
3DSSD	88.36	79.57	74.55	26.3	2020	[167]
SASSD	88.75	79.79	74.16	24.9	2020	[185]
Pyramid RCNN	87.03	80.30	76.48	8.9	2021	[186]
ST3D	-	-	74.61	-	2021	[187]
SASA	88.76	82.16	77.16	27.8	2022	[188]
PointDistiller	88.10	76.90	73.80	-	2023	[189]
DCGNN	89.65	79.80	74.52	9.0	2023	[190]
Projection-based						
DeepthCN	37.59	23.21	18.01	-	2017	[168]
RT3D	72.85	61.64	64.38	11.2	2018	[169]
BirdNet	88.92	67.56	68.59	9.1	2018	[170]
PIXOR	81.70	77.05	72.95	10.8	2018	[191]
Complex-YOLO	67.72	64.00	63.01	59.4	2018	[192]
BirdNet+	70.14	51.85	50.03	10.0	2020	[171]
E2E-PL	79.60	58.80	52.10	-	2020	[173]
Pseudo-L	23.74	17.74	15.14	-	2022	[174]
Ri-Fusion	85.62	75.35	68.31	26.0	2023	[193]
Voxel-based						
3DFCN	84.20	75.30	68.00	-	2017	[194]
VoxelNet	77.47	65.11	57.73	30.3	2018	[175]
Second	83.13	73.66	66.20	20.0	2018	[195]
PV-RCNN	90.25	81.43	76.82	12.5	2020	[177]
HVNet	87.21	77.58	71.79	31.3	2020	[196]
TANet	83.81	75.38	67.66	28.8	2020	[197]
Voxel RCNN	90.09	81.62	77.06	25.0	2021	[176]
MA-MFFC	92.60	84.98	83.21	7.1	2022	[180]
PDV	90.43	81.86	77.49	7.4	2022	[181]
SAT-GCN	79.46	86.55	78.12	8.2	2023	[182]
BSAODet	88.89	81.74	77.24	-	2023	[198]

**Table 5:** Comparative analysis of different sensors. Numbers “1”–“5” denote the level from extremely low to low, medium, high, and extremely high, respectively.

Sensor	Camera	Radar	LiDAR
Silhouette Representation	5	2	3
Color Perception	5	1	1
Velocity Measurement	2	5	2
Angle Resolution	5	3	4
Range Resolution	2	4	5
Object Detection	5	3	4
Object Classification	5	1	3
Field of View	3	4	4
Adaptability to Complex Weather	2	5	2
Sensor Size	2	2	4
Cost	3	1	5

**Table 6:** Performance of some of the latest radar–camera-based models on the nuScenes dataset.

Model	Metrics							FPS	Year	Reference
	mAP	NDS	mATE	mASE	mAOE	mAVE	mAAE			
CenterFusion	32.6	44.9	63.1	26.1	51.6	61.4	11.5	-	2021	[238]
CRAFT	41.1	52.3	46.7	26.8	45.3	51.9	11.4	4.1	2023	[231]
RCBEV	40.6	45.6	48.4	25.7	58.7	70.2	14.0	-	2023	[234]
MVFusion	45.3	51.7	56.9	24.6	37.9	78.1	12.8	-	2023	[239]
CRN	57.5	62.4	46.0	27.3	44.3	35.2	18.0	7.2	2023	[240]

**Table 7:** Performance overview of LiDAR–camera-based models on KITTI dataset.

Model	Fusion	Car AP_3D_ (IoU = 0.7)			FPS	Year	Reference
		Easy	Moderate	Hard			
MV3D	Feature	-	-	-	2.8	2017	[241]
AVOD-FPN	Feature	81.94	71.88	66.38	10.0	2018	[242]
PointFusion	Feature	77.92	63.00	53.27	0.8	2018	[252]
ContFuse	Feature	82.54	66.22	64.04	16.7	2018	[253]
F-PointNet	Decision	83.76	70.92	63.65	-	2018	[243]
IPOD	Decision	84.10	76.40	75.30	-	2018	[254]
MMF	Feature	86.81	76.75	68.41	12.5	2019	[255]
F-ConvNet	Decision	85.88	76.51	68.08	-	2019	[256]
SIFRNet	Feature	85.62	72.05	64.19	-	2020	[257]
PointPainting	Feature	92.45	88.11	83.36	-	2020	[258]
EPNet	Feature	88.94	80.67	77.15	-	2020	[259]
F-PointPillars	Feature	88.90	79.28	78.07	14.3	2021	[260]
Fast-CLOCs	Feature	89.11	80.34	76.98	13.0	2022	[261]
SFD	Feature	91.73	84.76	77.92	-	2022	[262]
VPFNet	Feature	91.02	83.21	78.20	10.0	2022	[263]
FocalsConv	Feature	92.26	85.32	82.95	6.3	2022	[264]
VFF	Feature	92.31	85.51	82.92	-	2022	[265]
EPNet++	Feature	91.37	81.96	76.71	-	2022	[266]
PA3DNet	Feature	90.49	82.57	77.88	47.6	2023	[267]
DVF-PF	Feature	90.99	82.40	77.37	-	2023	[268]
LoGoNet	Feature	92.04	85.04	84.31	-	2023	[269]
VirConvNet	Feature	95.81	90.29	88.10	10.9	2023	[249]
VoxelNextFusion	Feature	90.40	82.03	79.86	18.5	2024	[270]

**Table 8:** Autonomous driving sensor solutions of some manufacturers.

Company	Autonomous Driving System	Sensor Settings	Link
Baidu	Apollo	13 cameras 5 mmWave radars 2 LiDAR	https://www.apollo.auto/ (accessed on 12 May 2024)
Tesla	Autopilot	8 cameras mmWave radars 12 ultrasonic radars	https://www.tesla.com/ (accessed on 12 May 2024)
Waymo	Waymo Driver	29 cameras 6 mmWave radars 4 LiDAR	https://waymo.com/ (accessed on 12 May 2024)
NIO	Aquila	11 cameras 4 mmWave radars 1 LiDAR 12 ultrasonic radars	https://www.nio.cn/ (accessed on 12 May 2024)
Xpeng	XPILOT	13 cameras 12 ultrasonic radars 5 mmWave radars	https://www.xiaopeng.com/ (accessed on 12 May 2024)

## Data Availability

The original data presented in the study are openly available: the pictures in Figure 1 were derived from Cityscapes [16]; the pictures in Figure 4 were sourced from nuScenes [27]; Figure 2, Figure 7, Figure 8, Figure 9 and Figure 10 display pictures obtained from KITTI [15].
